# VGAE-CCI: variational graph autoencoder-based construction of 3D spatial cell–cell communication network

**DOI:** 10.1093/bib/bbae619

**Published:** 2024-11-24

**Authors:** Tianjiao Zhang, Xiang Zhang, Zhenao Wu, Jixiang Ren, Zhongqian Zhao, Hongfei Zhang, Guohua Wang, Tao Wang

**Affiliations:** College of Computer and Control Engineering, Northeast Forestry University, Harbin 150040, China; College of Computer and Control Engineering, Northeast Forestry University, Harbin 150040, China; College of Computer and Control Engineering, Northeast Forestry University, Harbin 150040, China; College of Computer and Control Engineering, Northeast Forestry University, Harbin 150040, China; College of Computer and Control Engineering, Northeast Forestry University, Harbin 150040, China; College of Computer and Control Engineering, Northeast Forestry University, Harbin 150040, China; College of Computer and Control Engineering, Northeast Forestry University, Harbin 150040, China; Faculty of Computing, Harbin Institute of Technology, Harbin 150001, China; School of Computer Science, Northwestern Polytechnical University, Xi'an 710072, China

**Keywords:** cell–cell communication, scRNA-seq, ST-seq, variational graph autoencoder

## Abstract

Cell–cell communication plays a critical role in maintaining normal biological functions, regulating development and differentiation, and controlling immune responses. The rapid development of single-cell RNA sequencing and spatial transcriptomics sequencing (ST-seq) technologies provides essential data support for in-depth and comprehensive analysis of cell–cell communication. However, ST-seq data often contain incomplete data and systematic biases, which may reduce the accuracy and reliability of predicting cell–cell communication. Furthermore, other methods for analyzing cell–cell communication mainly focus on individual tissue sections, neglecting cell–cell communication across multiple tissue layers, and fail to comprehensively elucidate cell–cell communication networks within three-dimensional tissues. To address the aforementioned issues, we propose VGAE-CCI, a deep learning framework based on the Variational Graph Autoencoder, capable of identifying cell–cell communication across multiple tissue layers. Additionally, this model can be applied to spatial transcriptomics data with missing or partially incomplete data and can clustered cells at single-cell resolution based on spatial encoding information within complex tissues, thereby enabling more accurate inference of cell–cell communication. Finally, we tested our method on six datasets and compared it with other state of art methods for predicting cell–cell communication. Our method outperformed other methods across multiple metrics, demonstrating its efficiency and reliability in predicting cell–cell communication.

## Introduction

Cell–cell communication plays a crucial role in maintaining normal biological functions, regulating development and differentiation, and controlling immune responses [1–3]. Cell–cell communication is typically mediated by various membrane-bound factors, such as ligands, receptors, extracellular matrix, integrins, and junctional proteins [4]. The life of multicellular organisms depends on the coordination of cellular activities [5], and this coordination is reliant on cell–cell interactions (CCI) between different cell types [6, 7] and tissues within the organism. Therefore, research on cellular functions increasingly needs to consider the spatial environment of each cell [3, 8].

In recent years, several computational strategies based on ligand-receptor (L-R) gene pairs have been developed to identify CCI from single-cell RNA sequencing (scRNA-seq) data, such as SingleCellSignalR [9], iTALK [10], CellPhoneDB [11], and CellChat [12]. With the rapid advancement of scRNA-seq and spatial transcriptomics sequencing (ST-seq) technologies, research on cell–cell communication is becoming more in-depth and comprehensive [13, 14]. These advanced technologies allow for the analysis of gene expression and spatial organization at single-cell resolution, revealing the complex interactions between different cell types and subtypes [15–19]. scRNA-seq enables the precise identification of the transcriptomic features of individual cells, uncovering cellular heterogeneity and dynamic changes [20], while ST-seq provides information on the spatial location of cells in their original tissue environment, preserving the tissue structure and the spatial relationship between cells [21–23]. By combining these two technologies, researchers can construct more comprehensive and accurate cell–cell communication networks.

However, ST-seq data often contain incomplete data and systematic biases. Due to changes in sample processing and sequencing, data inaccuracies and incomplete data can occur [24–26]. Therefore, there is a need for a method that can accurately identify cell–cell communication from missing or partially incomplete data to improve the precision and reliability of cell–cell communication studies. Current methods often predict cell–cell communication within a single tissue layer, ignoring the linkages between multiple tissue layers [27, 28]. However, many diseases involve interactions across multiple tissue layers. Predicting cell–cell communication across multi-layered tissue sections can help identify key regulatory nodes and communication networks of diseases, revealing the mechanisms of disease onset, progression, and development, thereby providing new perspectives for disease diagnosis and treatment.

In this study, we developed VGAE-CCI, a deep learning framework based on VGAE, designed to identify CCI from scRNA-seq and ST data. This deep learning framework can not only extract latent features related to CCIs from spatial transcriptomics data with missing or partially incomplete data, thereby reducing noise, reconstructing existing interactions, and recovering missing interactions to overcome the limitations of incomplete data. Additionally, it can accurately identify cell–cell communication across multi-layered tissues, exploring connections between cells in different tissue layers. To validate the practicality of VGAE-CCI, we demonstrated its overall functionality using six publicly available scRNA-seq and ST datasets. Experimental results showed that VGAE-CCI outperformed other methods in metrics such as area under the receiver operating characteristic curve (AUROC) and Accuracy (ACC). This clearly demonstrates that our approach can reconstruct existing interactions, recover missing interactions from datasets with missing or partially incomplete data, and identify cell–cell communication across multi-layered tissue sections.

## Materials and Methods

### Workflow of VGAE-CCI

VGAE-CCI combines VGAE and adversarial networks to learn from single-cell and spatial transcriptomics data, generating latent distributions that capture the intrinsic associations between CCIs and gene expression patterns. The role of the adversarial regularization module is to improve the model's robustness against input perturbations and noise. In graph data, adversarial regularization helps VGAE maintain stable performance when facing minor perturbations in graph structures or node features, such as data noise or missing data. Simultaneously, it encourages the latent representations generated by the encoder to be closer to the true distribution.

**Figure 1:**
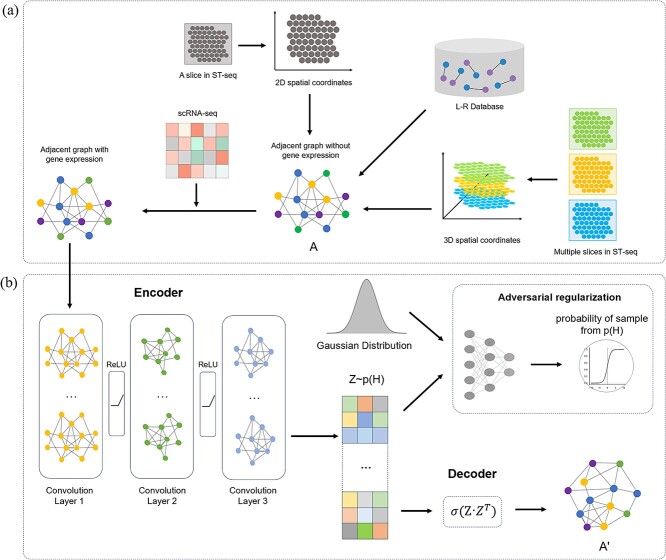
Workflow of VGAE-CCI. VGAE-CCI consists of an encoder, a decoder, and an adversarial regularization module within the VGAE framework. The encoder is composed of three layers of GCN, and the decoder is a Sigmoid function applied to the dot product of the latent variables. (a) VGAE-CCI integrates scRNA-seq data and the cellular adjacency graph to construct a cell adjacency network. (b) The encoder of VGAE-CCI is composed of three layers of GCN, which receives the cell adjacency network graph and generates a new matrix A', representing the reconstructed CCI network.

VGAE-CCI first constructs an undirected adjacency network of cells using the SCTDB (See Data Processing section) database or the spatial coordinates of cells, where nodes represent cells (or genes) and edges represent adjacent cell pairs (or L-R pairs), as shown in Fig. 1(a). This network is represented by an adjacency matrix *A*, with single-cell gene expression data serving as the features of the nodes in the adjacency matrix. Subsequently, we input this adjacency network with node features into a neural network composed of three GCNs, as illustrated in Fig. 1(b). In the GCN, the generated latent representation *Z* captures the feature information of individual cells and their neighboring cells, with *Z* constrained by an adversarial regularization module derived from a prior Gaussian distribution. Based on this latent representation, the decoder generates the reconstructed CCI network adjacency matrix *A'* through a dot product operation, which shows the reconstructed CCI network. However, to achieve cross-tissue cell–cell communication analysis, the ST-seq and scRNA-seq data need to be aligned using the PASTE [29] method. After alignment, we obtain three-dimensional spatial coordinates and a three-dimensional spatial structure map. Next, we input these three-dimensional coordinates and spatial structure maps, along with the gene expression matrix, into the GCN to reconstruct the three-dimensional cell–cell communication structure.

### Construction of adjacency matrices

When constructing the adjacency matrix, we provide two methods. When the goal is to explore cell-level communication, the spatial coordinates of cells are required to construct the adjacency matrix. Conversely, when exploring gene-level communication, the adjacency matrix can be constructed using the real SCTDB database we provide.

For exploring cell-level communication, we offer the following method to construct the adjacency matrix (similar to DeepLinc [30]). From a general biological perspective, it is reasonable to assume that in solid tissues, most cells can directly contact three or more other cells, and it is generally assumed that a cell interacts with its neighboring cells. Based on this assumption, we can construct the adjacency matrix. First, we calculate the distance matrix using the spatial coordinates of the cells. Since most other methods use Euclidean distance to calculate the distance between two cells, for ease of comparison with them, we also use Euclidean distance. Here, the Euclidean distance is used to compute the distance between cells:


$$ {D}_{\left(i,j\right)}=\sqrt{\sum_{k=1}^d{\left({x}_{ik}-{x}_{jk}\right)}^2} $$

Here, ${D}_{\left(i,\kern0.5em j\right)}$is the distance between cell *i* and cell *j*, ${x}_{ik}$ and ${x}_{jk}$ are the coordinates of cell *i* and cell *j* in the *k*th dimension.

Next, we determine the nearest neighboring cells for each cell *i*. For each cell *i*, we find its *n* nearest neighboring cells and store these neighboring cells in ${N}_i$:


$$ {N}_i=\left\{ the\ smallest\ n\ elements\ in\ {D}_i\right\} $$

Here, ${D}_i$ represents the *i*-th row of the distance matrix *D*.

Then, the adjacency matrix is constructed:


$$ {A}_{ij}=\left\{\begin{array}{@{}ll}1,& \mathrm{if}\ \mathrm{j}\in{N}_i\\{}\ 0, & \mathrm{otherwise}\end{array}\right. $$

Finally, we need to symmetrize the constructed adjacency matrix to make it a symmetric matrix.

When exploring gene-level communication, the following method can be used to construct the adjacency matrix. Given gene expression data $E=\left\{{e}_1,{e}_2,\dots, {e}_u\right\}$ where ${e}_i$ is the identifier of the *i*-th gene and *u* is the total number of genes, an adjacency matrix *A* can be constructed by combining the curated L-R pairs. If genes *i* and *j* from the gene expression matrix are present and correspond in the SCTDB, then ${e}_i$ and ${e}_j$ are considered to be related, and the adjacency matrix ${A}_{ij}$ can be represented as follows:


$$ {A}_{ij}=\left\{\begin{array}{@{}ll} 1, & if\ {e}_i\ and\ {e}_j\ are\ related\\{}0, & otherwise\end{array}\right. $$

Similarly, the constructed adjacency matrix should also be symmetrized.

### Construction of cell communication models

We constructed a cell–cell communication network model based on VGAE. By combining GCN with VAE, an end-to-end model can be built to simultaneously learn the graph structural information and latent representations from the cell–cell communication network. The integration of these two components enhances the model's representation and generation capabilities for cell–cell communication networks. Moreover, since cell–cell communication networks are typically sparse and high-dimensional, the combination of GCN and VAE can effectively handle such data, improving the model's robustness and generalization ability.

VAE is a generative model that can learn latent representations of data and generate new samples. In the context of cell–cell communication networks, this can be used to generate potential ligand-receptor pairs and simulate new cell–cell communication networks. GCN, as the encoder, encodes the data *x* to obtain the mean ${\mu}_{\varnothing }(x)$ and standard ${\sigma}_{\varnothing }(x)$ deviation of the latent variable *z*:


$$ {q}_{\varnothing}\left(z|x\right)=\mathcal{N}\left(z:{\mu}_{\varnothing }(x),{\sigma}_{\varnothing }{(x)}^2\right) $$

Here, ${\mu}_{\varnothing}\left(\mathrm{x}\right)$ and ${\sigma}_{\varnothing}\left(\mathrm{x}\right)$ are learned through neural network parameters $\varnothing$. To allow gradients to pass through the random variable *z*, the reparameterization trick is used:


$$ z={\mu}_{\phi }(x)+{\sigma}_{\phi }(x)\odot \epsilon $$

Here, $\epsilon \sim \mathcal{N}\left(0,I\right)$ is the noise sampled from a standard normal distribution.

The loss function includes a reconstruction loss and a KL divergence loss. The reconstruction loss is used to measure the difference between the generated data and the original data. Here, we use the cross-entropy loss:


$$ {\mathcal{L}}_{\mathrm{recon}}=-{\mathbb{E}}_{q_{\phi}\left(z|x\right)}\left[\log{p}_{\theta}\left(x|z\right)\right] $$

Here, ${p}_{\theta}\left(x|z\right)$ represents the conditional probability distribution of generating data *x* given the latent variable *z*.

The KL divergence loss is used to measure the difference between the approximate posterior distribution ${q}_{\varnothing}\left(z|x\right)$ and the prior distribution $p\left(\mathrm{z}\right)$:


$$ {\mathcal{L}}_{\mathrm{KL}}={D}_{\mathrm{KL}}\left({q}_{\phi}\left(z\mid x\right)\parallel p(z)\right)=\frac{1}{2}{\sum}_{j=1}^d\left(1+\log \left({\sigma}_j^2\right)-{\mu}_j^2-\sigma \right) $$

The total loss function is the weighted sum of the reconstruction loss and the KL divergence loss:


$$ \mathcal{L}={\mathcal{L}}_{\mathrm{recon}}+\beta{\mathcal{L}}_{\mathrm{KL}} $$

Here, $\beta$ is a weight parameter used to balance the reconstruction loss and the KL divergence loss, which is set to 1 by default.

The GCN, used as the encoder, aggregates features based on neighboring nodes, updating the target node through weighted contributions from neighboring nodes. The propagation between layers in the GCN neural network is as follows:


$$ {H}^{\left(l+1\right)}=\sigma \left({\overset{\sim }{D}}^{-1/2}\overset{\sim }{A}{\overset{\sim }{D}}^{-1/2}{H}^{(l)}{W}^{(l)}\right) $$

In the formula, $\overset{\sim }{A}=A+I$ is the identity matrix, *A* is the adjacency matrix of the nodes, and $\overset{\sim }{D}$ is the degree matrix of $\overset{\sim }{A}$. The formula is given by ${\overset{\sim }{D}}_{ii}=\sum_j\kern0.1em {\overset{\sim }{A}}_{ij}$.*H* represents the features at each layer, and $\sigma$ is the activation function.

This propagation method allows parameters to be shared across all nodes, which means that the update rules are the same for each node. This method is more suitable for identifying all genes in the network and classifying them according to their expression values, which is more in line with the needs.

In our model, features are extracted through convolutional layers, and latent space representations are generated using the reparameterization trick. For the first layer of graph convolution:


$$ {H}_{(1)}= ReLU\left(\hat{A}X{W}^{(0)}\right) $$

The *l*-th layer of graph convolution is given by:


$$ {H}_{(l)}= ReLU\left(\hat{A}{H}^{\left(l-1\right)}{W}^{\left(l-1\right)}\right) $$

Next, compute the mean and standard deviation of the latent space:


$$ \mu =\hat{A}{H}^{(l)}{W}^{(l)} $$

$$ \log \sigma =\hat{A}{H}^{(l)}{W}^{\left(l+1\right)} $$

For the reparameterization method,


$$ Z=\mu +\varepsilon \cdotp \exp \left(\log \sigma \right) $$

Here, $\epsilon \sim \mathcal{N}\left(0,I\right)$. Finally, we use the dot product decoder to compute the reconstructed adjacency matrix:


$$ \hat{A}=\sigma \left(Z{Z}^T\right) $$

Here, $\sigma$ represents the sigmoid function.

### Alignment of multilayer tissue layers

In order to analyze cell–cell communication between multi-layered tissue sections, the most critical step is to align these sections for subsequent analysis and processing. There are many ways to align tissue sections with each other, but some require high-quality image data and some require a priori manual annotation of information. Here, we use the PASTE method because it takes into account both gene expression levels and location information. This method can effectively integrate multiple sections on the basis of maintaining gene expression and spatial location information, and the method can process sections of different sizes and shapes, which is flexible. Compared with other algorithms, this algorithm can better handle the noise and variability in the data, thereby increasing the robustness of alignment, and the algorithm is more suitable for complex spatial transcriptomics data.

First, we extract feature vectors from each layer, typically constructed using gene expression data or coordinates for each cell. Next, we calculate the distance between each pair of cells, using Euclidean distance to compute the cell–cell distance. Let ${x}_i$ and ${x}_j$ are the feature vectors of two cells; their Euclidean distance ${d}_{ij}$ is calculated as follows:


$$ {d}_{ij}=\sqrt{\sum_{k=1}^n\kern0.1em {\left({x}_{ik}-{x}_{jk}\right)}^2} $$

Here, ${x}_{ik}$ and ${x}_{jk}$ are the *k*th components of the feature vectors.

Next, align the layers by optimizing the alignment objective function. First, initialize the matching matrix *P*:


$$ P=\frac{1}{n\times m} $$

Here, *n* and *m* represent the number of points in the two layers.

Then, for each layer, compute the distance matrix between its points:


$$ {D}_X\left(i,j\right)=\parallel{X}_i-{X}_j\parallel $$

$$ {D}_Y\left(k,l\right)=\parallel{Y}_k-{Y}_l\parallel $$

Here, ${D}_X\left(i,j\right)$ and ${D}_Y\left(k,l\right)$ are the distance matrices of the two cell graphs, and *X* and *Y* are the point sets of the two layers, respectively. The PASTE algorithm uses the Gromov–Wasserstein (GW) distance to align the two cell graphs. The GW distance measures the discrepancy between the distance matrices of the cells, aiming to minimize the GW distance between the two cell graphs. The optimization objective function can be expressed as follows:


$$ C\left(i,k,j,l\right)={\left({D}_X\left(i,j\right)-{D}_Y\left(k,l\right)\right)}^2 $$

$$ GW\left(C,P\right)=\sum_{i,j,k,l}\kern0.1em C\left(i,k,j,l\right)P\left(i,k\right)P\left(j,l\right) $$

Iteratively optimize and update the matching matrix *P* using the Sinkhorn-Knopp algorithm.


$$ P\leftarrow \operatorname{diag}(u)\cdotp P\cdotp \operatorname{diag}(v) $$

Here, *u* and *v* are normalization vectors, and *P* is iteratively updated to satisfy row and column sum constraints.

Finally, generalized Procrustes analysis is used to optimize rotation and scaling parameters, enhancing the accuracy of the alignment results:


$$ R,t=\arg \underset{R,t}{{min}}\parallel RX+t-Y{\parallel}_F^2 $$

Here, *R* is the rotation matrix and *t* is the translation vector. Through the above steps, multi-layer tissue layers can be successfully aligned.

### Data processing

Accurately predicting biologically meaningful cell–cell communication requires a comprehensive set of signaling L-R pairs. In recent years, several published L-R pair databases have emerged, such as CellChatDB, CellTalkDB [31], CellCallDB [23], CellPhoneDB, connectomeDB2020 [32], and iTALK, among others. These databases have been developed to better predict CCI by providing literature-supported L-R pairs for human and mouse.

To better predict cell–cell communication, we integrated publicly available ligand-receptor pair databases to construct SCTDB. SCTDB includes human and mouse L-R interactions. To ensure that the data in SCTDB is based on reliable and validated scientific studies, we prioritized interactions supported by literature to enhance the accuracy and reliability of predicting cell–cell communication. Additionally, we considered the presence of multi-subunit complexes, which are crucial functional units involved in complex L-R interactions during cellular processes. By accounting for these factors, we ensured that the predicted interactions reflect the complexity of actual biological processes. Finally, we integrated all the data and removed redundant L-R pairs to ensure the uniqueness and accuracy of the dataset. As a result, SCTDB retained 5786 human-validated L-R interactions and 4806 mouse-validated L-R interactions (Supplementary Data S1 and S2 available online at http://bib.oxfordjournals.org/).

The VGAE-CCI framework takes the scRNA-seq gene expression matrix and the corresponding adjacency matrix as primary inputs. For the gene expression matrix, we first perform data cleaning by removing cells and genes with all-zero values as an initial filtering step. Next, we standardize the filtered gene expression matrix to reduce data bias and improve analysis accuracy. The adjacency matrix can be obtained through two methods: one approach involves obtaining cell coordinate data and constructing the adjacency matrix as referenced in the methods section; the other approach is based on real ligand-receptor pairs. Using SCTDB, we match the processed gene expression matrix with SCTDB, extract matching genes to construct a symmetric matrix, and finally build the adjacency matrix based on the L-R pairs.

### Evaluation criteria for the model

Here, we use Area Under the Curve (AUC), Average Precision (AP), and ACC to evaluate the performance of VGAE-CCI in CCI prediction. These metrics are well-known in the field of bioinformatics and have been widely used, defined as follows:


$$ \mathrm{AUC}={\sum}_{i=1}^{n-1}\left( FP{R}_{i+1}-{FPR}_i\right)\times \frac{TP{R}_{i+1}+ TP{R}_i}{2} $$

where *FPR_i_* and *TPR_i_* are the False Positive Rate and True Positive Rate at the *i*th point:


$$ \mathrm{AP}=\sum_n\kern0.1em \left({R}_n-{R}_{n-1}\right){P}_n $$

where *P_n_* is the precision at the *n*th threshold, *R_n_* is the recall at the *n*th threshold, and *R_n-1_* is the recall at the previous threshold.


$$ A\mathrm{CC}=\frac{TP+ TN}{TP+ TN+ FP+ FN} $$

where *TP* is the number of true positives, *FN* is the number of false negatives, *FP* is the number of false positives, and *TN* is the number of true negatives.

## Results

### Comparison of performance in inferring CCI with other methods

To evaluate the performance of VGAE-CCI, we compared it with six other methods (DeepCCI [33], CellPhoneDB, SingleCellSignalR, CellChat, NATMI, DeepLinc) on three different datasets. Each method used its default parameters to infer CCI. For each dataset, the results obtained by our method are obtained under 30 epochs and averaged based on 30 runs under different seeds.

The human colorectal cancer (CRC) liver metastasis spatial transcriptomics dataset is a comprehensive resource for studying high-throughput spatial gene expression during the liver metastasis of colorectal cancer. By analyzing CCIs within this dataset, we can gain deeper insights into the spatial distribution of cancer cells in the liver, their migration pathways, and their interactions with other cell types in the microenvironment. This is crucial for understanding the complex dynamics of the tumor microenvironment, the mechanisms of cancer cell metastasis, and potential therapeutic targets. Therefore, we compared the performance of VGAE-CCI with six other methods on this dataset. Each method used its default threshold, and performance was evaluated using ACC. The accuracy is calculated based on SCTDB. As shown in Table 1, VGAE-CCI achieved the highest ACC value.

**Table 1:** Comparison of ACC between VGAE-CCI and six other methods on two real datasets.

	VGAE-CCI	DeepCCI	CellPhoneDB	SingleCellSignalR	CellChat	NATMI	DeepLinc
CRC	**0.84**	0.795	0.731	0.724	0.803	0.731	0.56
Melanoma	**0.831**	0.79	0.744	0.755	0.785	0.754	0.635

Next, we compared VGAE-CCI with other methods on the human melanoma dataset. Similarly, we used ACC for performance evaluation. As shown in Table 1, VGAE-CCI achieved the highest ACC value. These results indicate that VGAE-CCI is highly effective in identifying relevant L-R pairs in cancer datasets and demonstrates high accuracy.

**Figure 2:**
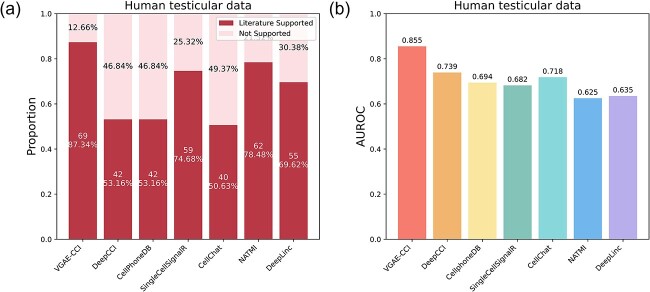
Comparison of VGAE-CCI with other methods. (a) The number of literature-supported CCIs predicted by each method using their default thresholds. (b) Comparison of AUROC between VGAE-CCI and other CCI prediction methods on the human testicular dataset.

Finally, we compared the performance of VGAE-CCI with six other CCI prediction methods on the human testicular dataset. Each method used its default threshold. VGAE-CCI identified 69 literature-supported cell–cell communications from Sertoli cells to SSCs, while DeepCCI, CellPhoneDB, SingleCellSignalR, CellChat, NATMI, and DeepLinc identified 42, 42, 59, 40, 62, and 55 literature-supported CCIs, respectively. Reportedly, 87.34% of the literature-supported CCIs identified by VGAE-CCI (69/79) are involved in spermatogenesis (see Supplementary Data S3 available online at http://bib.oxfordjournals.org/). The identification results from DeepCCI, CellPhoneDB, SingleCellSignalR, CellChat, NATMI, and DeepLinc are shown in Fig. 2(a). We then used AUROC to compare these methods, with the results shown in Fig. 2(b). VGAE-CCI achieved the highest AUROC. These results suggest that VGAE-CCI may provide more accurate inference of cell–cell communications compared to others methods and has the capability to identify biologically relevant CCIs from scRNA-seq data.

### Performance of VGAE-CCI on noise datasets

Single-cell spatial transcriptomics analysis provides snapshots of the cellular interaction landscape, which are likely only a small subset of the complete cellular interaction network and are often contaminated by high noise. Therefore, VGAE-CCI is crucial as it efficiently learns from the information provided by cellular spatial organization and single-cell transcriptomics analysis, filters out noise, accurately infers potential interactions, and ultimately reconstructs the complete cellular interaction landscape. This is a key benchmark.

**Figure 3:**
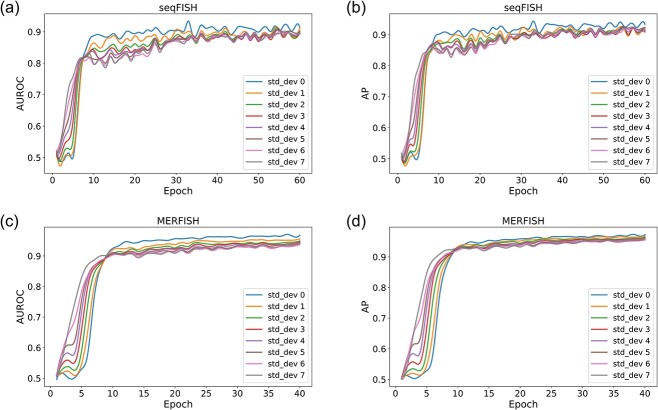
Performance of VGAE-CCI under different noise conditions. (a) AUROC curves for seqFISH dataset with Gaussian noise of varying standard deviations. (b) Precision curves for VGAE-CCI on the seqFISH dataset. (c) AUROC values under different noise levels for the MERFISH dataset. (d) Precision values for VGAE-CCI on the MERFISH dataset.

VGAE-CCI was applied to two spatial transcriptomics datasets, seqFISH and MERFISH. To assess the robustness of VGAE-CCI to artificial noise, different levels of Gaussian noise were added to the original gene expression data. As shown in Fig. 3, on the two datasets, it can be observed that under the condition of Gaussian noise with a standard deviation of 0–7, the AUROC and AP of VGAE-CCI increase rapidly within the first 10 epochs, and then the growth rate gradually flattens. Additionally, regardless of the noise standard deviation, VGAE-CCI's AUROC and AP values eventually exhibited high consistency. Therefore, VGAE-CCI demonstrates strong resistance to interference, with AUROC and AP remaining relatively stable across different levels of noise (the std_dev represents the magnitude of the standard deviation of the noise). This indicates that VGAE-CCI has significant robustness to noise in gene expression data, which is crucial for handling high-noise spatial transcriptomics data.

### Performance of VGAE-CCI on incomplete datasets

**Figure 4:**
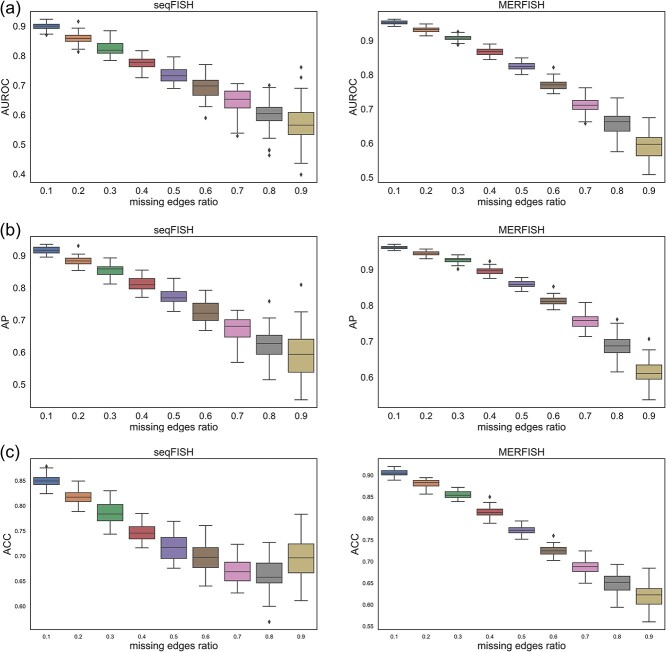
Performance of VGAE-CCI in reconstructing CCI networks. (a) Boxplots of AUROC over 50 iterations for varying proportions of randomly removed existing edges in both seqFISH and MERFISH datasets. (b) Values of AP for different proportions of removed existing edges. (c) Prediction accuracy of VGAE-CCI for varying proportions of removed edges.

Next, we evaluated the performance of VGAE-CCI in recovering partially missing CCI networks. Specifically, we randomly removed varying proportions of existing edges from the original CCI network and then used the remaining network and single-cell transcriptomic data to train the VGAE-CCI model. By calculating the values of AUROC, AP, and ACC, we assessed the performance of VGAE-CCI in identifying the interactions that were randomly removed at different proportions. As shown in Fig. 4, VGAE-CCI demonstrated high accuracy in imputing the missing interactions. Even when half of the interactions were missing from the input, VGAE-CCI was still able to recover these missing edges with 75–80% accuracy. This result indicates that VGAE-CCI can effectively learn from limited and incomplete CCI networks and accurately recover the missing edges. This imputation and predictive capability of VGAE-CCI is crucial as it enables the inference of a complete CCI landscape from missing or partially missing spatial transcriptomic data.

In summary, VGAE-CCI demonstrated a high level of robustness and recovery capability in the context of partially missing CCI networks within gene expression data. Even in cases where a significant portion of interactions were lost, VGAE-CCI was able to maintain high AUROC, AP, and ACC, highlighting its effectiveness in handling complex and incomplete spatial transcriptomics data.

### VGAE-CCI identifies cell–cell communication in three-dimensional space

In a real physiological environment, cell–cell communication typically occurs in a three-dimensional structure. When analyzing single-layer tissue layers, there is a risk of missing interlayer cell–cell communication information, making it crucial to identify interactions across multiple tissue layers. The complexity of this three-dimensional tissue architecture is essential for understanding the dynamic regulation of cell interactions and functions. Multi-layer tissue layers can more accurately simulate the true interactions of cells within an entire tissue, reflecting cellular behavior in complex physiological environments. Exploring cell–cell communication across multiple tissue layers requires the alignment of these layers as a prerequisite.

Tissue layers typically exhibit varying spatial structures, and without proper alignment, the positions of cells on these layers will no longer correspond to the actual structure of the biological tissue. This misalignment can distort the relative positions and spatial relationships between cells, potentially leading to an inaccurate reflection of the spatial dependency of cell–cell communication. Consequently, this may affect the accuracy and interpretation of communication patterns between cells. As shown in Table 2, the effects of aligning tissue layers before and after alignment are presented. It is evident that without proper alignment of the tissue layers, the accuracy of prediction results decreases, which may lead to misunderstandings of cell function, tissue structure, and biological processes, ultimately impacting research conclusions and clinical applications.

**Table 2:** Effects before and after aligning tissue sections.

		Seed 1	Seed 2	Seed 3	Seed 4	Seed 5	Average
Before alignment	ACC	0.837	0.823	0.835	0.834	0.83	0.832
	AUROC	0.897	0.87	0.894	0.892	0.896	0.89
	AP	0.865	0.852	0.865	0.853	0.865	0.86
After alignment	ACC	0.891	0.883	0.892	0.859	0.867	**0.878**
	AUROC	0.928	0.928	0.936	0.919	0.917	**0.926**
	AP	0.891	0.893	0.901	0.89	0.88	**0.891**

**Figure 5:**
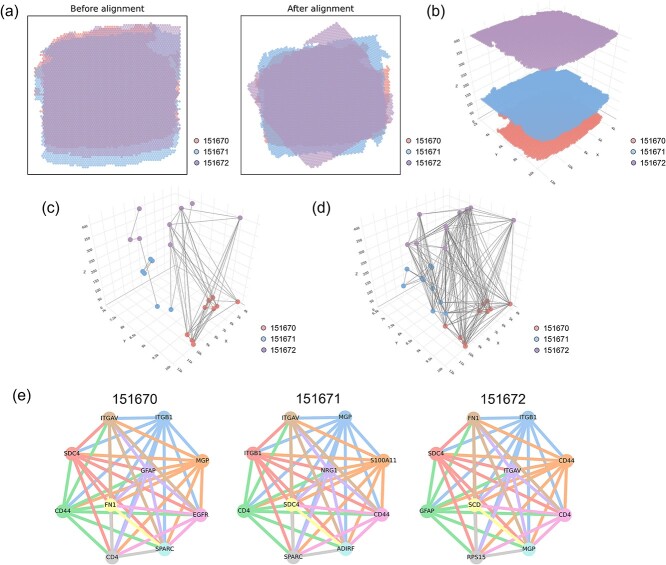
VGAE-CCI cross-tissue identification of cell–cell communication between 151 670, 151 671, and 151 672 tissue sections. (a) Comparison of multilayer tissue sections before and after alignment. (b) Visualization of the position of cells between the three layers of layers. (c) 3D diagram of the communication between top cells between different layers. (d) Cell–cell visualization with high probability of communication between layers. (e) Communication network diagram of the top 10 genes per layer.

Here, we utilized three tissue layers from the human dorsolateral prefrontal cortex dataset, labeled 151 670, 151 671, and 151 672. As shown in Fig. 5(a), the changes before and after alignment across the three layers of tissue layers are displayed. To further investigate cell–cell communication across different layers, we applied the data to the VGAE-CCI model. Figure 5(b), (c), and (d) presents the spatial positions of cells after alignment and the cell–cell communication views across the three layers of tissue layers. It is evident that cell–cell communication does not only occur within a single layer; some cells engage in communication across tissues with cells from other layers.

To further investigate the behavior of inter-layer communication, we visualized the communication networks of the top 10 genes within different layers, as shown in Fig. 5(e). The ITGAV (Integrin *av*) gene encodes an integrin protein that belongs to the integrin family. It is closely related to various cellular regulations, cell-matrix interactions, and biological processes such as skeletal system development, immune responses, and cell migration. Moreover, ITGAV is associated with the occurrence and development of multiple diseases, particularly cancer and autoimmune diseases. It plays a significant role in tumor metastasis, inflammatory responses, and platelet function. As illustrated in Fig. 5 (e), ITGAV is a gene shared among the three layers, yet the associated genes differ between layers. This indicates that VGAE-CCI can identify these relationships and discern the interactions and influences between different layers, thus exploring the mechanisms of inter-layer cell–cell communication. This understanding can contribute to better distinguishing between tumor tissues and normal tissues, which has positive implications for cancer treatment.

### Discovery of cancer-associated L-R pairs

**Figure 6:**
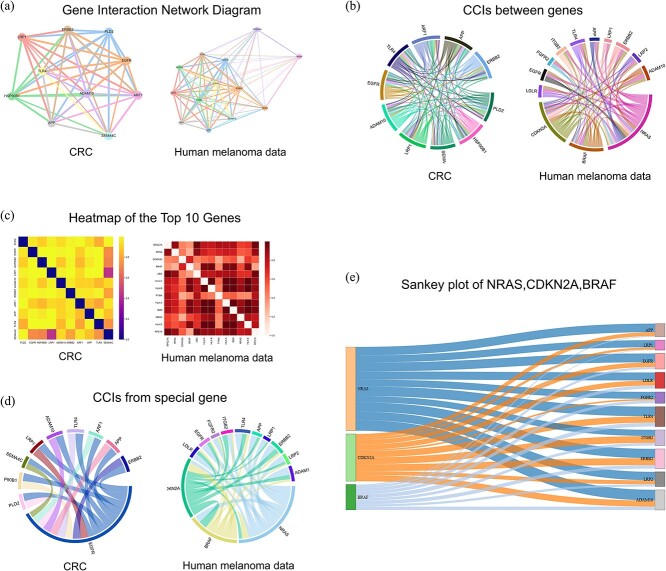
Visualization of VGAE-CCI. (a) Gene communication network diagrams on the Human Colorectal Cancer Liver Metastasis Dataset and the Melanoma Dataset. (b) Chord diagram representation of LR pair communication on two datasets. (c) Heatmap visualization of the TOP gene. (d) Communication chord diagram display of specific genes. (e) Sankey diagram of communication between NRAS, CDKN2A, BRAF genes, and TOP genes.

CRC refers to a malignant tumor that occurs in the colon or rectum, with high incidence and mortality rates globally. Liver Metastasis of CRC is one of the most common forms of metastasis in CRC, where cancer cells spread from the primary tumor site in the colon or rectum to the liver via the bloodstream or lymphatic system. In this study, we utilized spatial transcriptomics data of colorectal cancer liver metastasis to predict and analyze cell–cell communication. By employing advanced computational models, we inferred ligand-receptor interactions between different cells, identifying gene pairs with significant communication potential during liver metastasis. Figure 6(a)–(d) illustrates the network diagrams of the top 10 gene pairs with the highest communication probability as analyzed by VGAE-CCI in the context of colorectal cancer liver metastasis data. Through these network diagrams, we can visually observe the communication patterns of specific gene pairs within the liver metastasis microenvironment, providing crucial insights into the complex dynamics of the tumor microenvironment and identifying potential therapeutic targets. For instance, EGFR (Epidermal Growth Factor Receptor) is a gene that encodes the epidermal growth factor receptor, which is widely recognized for its significance in cancer. The role of EGFR in colorectal cancer liver metastasis is particularly critical. Its activation can initiate multiple intracellular signaling pathways, such as the PI3K/AKT, RAS/RAF/MEK/ERK, and JAK/STAT pathways, thereby influencing biological processes such as cell proliferation, apoptosis, angiogenesis, and migration. PLD2 (Phospholipase D2) is involved in processes like cell signaling, migration, invasion, and proliferation. PLD2 mediates intracellular signal transduction by generating the second messenger phosphatidic acid and interacts with various signaling molecules to regulate cellular physiological activities. EGFR and PLD2 interact in cell signaling to synergistically promote cell proliferation, migration, and invasion. Within the EGFR signaling pathway, PLD2 is considered one of the downstream effectors, enhancing the cell's response to EGFR-mediated growth factors by regulating key nodes within the EGFR signaling pathway. Their synergistic interaction plays a crucial role in tumor progression, and understanding this interaction is vital for developing new therapeutic strategies and overcoming drug resistance. Studies suggest that simultaneously targeting EGFR and PLD2 may enhance antitumor efficacy and overcome the issue of resistance associated with single-target therapies. Therefore, exploring the interaction mechanisms between EGFR and PLD2 in colorectal cancer liver metastasis is of great significance for cancer biology research and the formulation of clinical treatment strategies.

Melanoma is a malignant tumor originating from melanocytes, the pigment-producing cells of the skin. Although it accounts for only a small fraction of all skin cancers, melanoma is the most lethal, characterized by its highly aggressive and metastatic nature. In the human melanoma dataset, we visualized the L-R pairs involved in communication between different cells, as shown in Fig. 6(a)–(d). The development, prognosis, and treatment of melanoma are closely associated with mutations in genes such as BRAF, CKIT, and NRAS.NRAS mutations occur in approximately 20–25% of melanomas and contribute to signal transduction through pathways such as RAF-MEK-ERK and PI3K/Akt/mTOR, affecting cell growth, differentiation, and tumor development. The BRAF gene encodes the serine/threonine-protein kinase B-Raf, which is part of the MAPK/ERK signaling pathway involved in regulating cell division, differentiation, and apoptosis. The BRAF V600E mutation is the most common melanoma-related mutation, leading to constitutive activation of the B-Raf kinase, resulting in uncontrolled cell proliferation and tumor formation. The CDKN2A gene encodes two proteins: p16INK4a and p14ARF, both of which are tumor suppressors. p16INK4a inhibits cyclin-dependent kinases CDK4/6, while p14ARF stabilizes p53, together preventing cell cycle progression and promoting apoptosis. Mutations or deletions in the CDKN2A gene can result in the loss of function of p16 and p14, leading to the loss of cell cycle control, which promotes cell proliferation and tumor formation. As illustrated in Fig. 6(e), the communication relationships between BRAF, CDKN2A, NRAS, and other genes are depicted. In melanoma, BRAF and NRAS mutations promote cell proliferation by activating the MAPK/ERK pathway, while CDKN2A mutations further enhance tumor cell proliferation by removing inhibition of the cell cycle. When mutations in these three genes occur simultaneously, tumor cells gain a greater growth and survival advantage, leading to the rapid progression and worsening of melanoma. Therefore, understanding the interactions among these genes is crucial for the diagnosis and treatment of melanoma.

In conclusion, VGAE-CCI has played a pivotal role in elucidating the complex interactions and communication networks between cells in various cancer types, such as colorectal cancer liver metastasis and melanoma. By leveraging advanced computational models, VGAE-CCI can predict and visualize ligand-receptor interactions, identifying key genes that play crucial roles in the tumor microenvironment. This allows for a deeper understanding of cellular behavior and interactions, which is essential for discovering potential therapeutic targets and developing effective treatment strategies.

## Conclusion

ST-seq data often contain incomplete data and systematic biases, which can lead to inaccuracies and incomplete data. Previous methods may yield inaccurate predictions due to the presence of such noise. Moreover, these methods typically overlook intercellular communication across multiple tissue layers. In a real physiological environment, intercellular communication is not confined to a single tissue layer; rather, it is often three-dimensional, and a single tissue layer may miss communication information between layers. To address this, we propose a VGAE-based model, VGAE-CCI, in this paper, which can accurately identify intercellular communication from incomplete or partially incomplete data and predict communication between cells in three-dimensional space. Next, we applied VGAE-CCI to six real datasets, where it demonstrated high AUROC and ACC scores. Even when we manually introduced varying levels of Gaussian noise into the data, VGAE-CCI remained remarkably stable. This highlights VGAE-CCI's robustness to noise in gene expression data, which is crucial when dealing with high-noise spatial transcriptomic data. In summary, VGAE-CCI can accurately identify intercellular communication from noisy data and can detect communication between cells across multiple tissue layers. When applied to cancer datasets, VGAE-CCI can identify key genes that play critical roles in the tumor microenvironment. This deeper understanding of cellular behavior and interactions is essential for discovering potential therapeutic targets and developing effective treatment strategies.

**Key Points**

- VGAE-CCI can be applied to missing or partially incomplete spatial transcriptomics data and can more accurately infer cell–cell communication.
- VGAE-CCI can identify cell–cell communication across multi-layered tissue sections and can display a three-dimensional cell–cell communication map.
- VGAE-CCI exhibits excellent accuracy and robustness, making it suitable for various experimental designs, single-cell sequencing presumably derived from different organs, and cell communication of spatial transcriptomic data.

## Supplementary Material

Supplementary_Data_1_bbae619

Supplementary_Data_2_bbae619

Supplementary_Data_3_bbae619

## Data Availability

VGAE-CCI was benchmarked on six published datasets (Table 3), including the mouse visual cortex dataset analyzed using seqFISH, the mouse hypothalamic layer dataset analyzed using MERFISH technology, the human testis scRNA-seq dataset, the human colorectal cancer liver metastasis spatial transcriptomics dataset, the human melanoma dataset, and the human dorsolateral prefrontal cortex dataset analyzed using the Visium platform. The seqFISH dataset was downloaded from the data portal at the following URL: https://bitbucket.org/qzhu/smfish-hmrf/src/master/. The data were processed according to the procedures described in the original study, resulting in a final dataset containing 1597 cells and 125 genes. The MERFISH dataset was downloaded from https://datadryad.org/stash/dataset/10.5061/dryad.8t8s24818/, focusing on the mouse preoptic area. We used the layer region from animal number 18 at Bregma +0.11 mm, as this region contained the highest number of single cells. After removing ambiguous cells, the final dataset contained 4975 cells. The human testis scRNA-seq dataset comprised 3074 cells (GSE106487). The human CRC liver metastasis spatial transcriptomics dataset included four different cancer patients; here, we selected patient 1, which contained 2887 cells (GSE217414). The human melanoma dataset contained 4645 cells (GSE72056). The human dorsolateral prefrontal cortex (DLPFC) dataset analyzed using the Visium platform can be downloaded from the portal at the following URL: http://spatial.libd.org/spatialLIBD/. Source codes for the VGAE-CCI python packages and the related scripts are available at VGAECCI Github [https://github.com/zhangxiangz/VGAECCI]. Datasets summary.
